# Co-creating DigiPer mobile application: A digital peer support tool for managing mental, physical, and social health in serious mental illness

**DOI:** 10.1177/20552076251380648

**Published:** 2025-10-27

**Authors:** Jorunn Nærland Skjærpe, Hilde Marie Hunsbedt Fjellså, Karen L Fortuna, Bo Wang, Marianne Storm

**Affiliations:** 1 Department of Public Health, 56627 University of Stavanger, Stavanger, Norway; 2 Stavanger Municipality, Stavanger, Norway; 3 Department of Community and Family Medicine, 12285 Geisel School of Medicine at Dartmouth, Lebanon, NH, USA; 4 Faculty of Health Sciences and Social Care, 5562 Molde University College, Molde, Norway; 5 Department of Digital Health Services, Norwegian Centre for E-health Research, 60519 University Hospital of North Norway, Tromsø, Norway

**Keywords:** Co-creation, cultural adaptation, user involvement, peer support, digital mental health, mobile application, self-management, recovery

## Abstract

**Background:**

People with serious mental illness (SMI) often encounter physical health issues and persistent symptoms of mental illness. Digital tools and peer support can empower people with SMI to self-manage their mental, physical, and social health. This study aims to co-create and culturally adapt the DigiPer mobile application with service users, peer support workers, and Norwegian community mental healthcare professionals.

**Methods:**

We used a qualitative participatory design and principles of co-creation to develop DigiPer. We conducted workshops, videoconference demonstrations, and application testing with key stakeholders in the co-creation process. Written material from the co-creation process was transcribed and analyzed using reflexive thematic analysis.

**Results:**

Five themes and ten subthemes were developed. Theme (1) Feedback and challenges in DigiPer functioning, including usability in DigiPer and managing complexity in DigiPer classes. Theme (2) The peer support worker's role within DigiPer should include recognizing and addressing user challenges and balancing self-responsibility and support. Theme (3) The content of DigiPer addresses day-to-day challenges, supporting personal growth and goal setting, and DigiPer can be used as a tool to build supportive networks. Theme (4) DigiPer can encourage a healthy lifestyle, address substance uses and smoking habits and improve sleep and stress management. Theme (5) Provides a holistic view of health, connecting users to relevant services and addressing the need for support to overcome financial challenges.

**Conclusions:**

DigiPer demonstrated usability among Norwegian community mental healthcare stakeholders. It was perceived as relevant and functional and a promising tool to help people with SMI to self-manage their mental, physical, and social health. This study contributes to ongoing efforts to refine digital peer support interventions in Norway.

## Introduction

Research, technology, and understanding of mental illness are constantly evolving, necessitating innovations in the treatment of overall health.^1^ This evolution is particularly relevant for addressing the mental, physical, and social health needs of people with serious mental illness (SMI).^2–5^ By the term SMI, we refer to schizophrenia, schizoaffective disorder, psychotic disorders, major depressive disorder, and bipolar disorder.^6^ People with SMI are more likely to encounter physical health issues than the general population.^7^ A lack of focus on healthy lifestyles and self-management, as well as persistent symptoms of mental illness, are functional issues that often occur when people experience SMI.^2,8–10^ Moreover, social factors, such as difficulties related to psychosocial conditions and diminished social functioning, influence the prevalence and severity of SMI.^11–13^ Strategies to empower people with SMI to self-manage mental, physical, and social health are needed.^2,8–10^ Digital peer support interventions are strategies with the potential to empower people with SMI to self-manage their health.^5,14–17^

Digital health involves the use of information and communication technologies, including wearable devices, mobile health, telehealth, health information technology, and telemedicine, to manage health issues and promote well-being.^18^ In the current study, we use the term “digital peer support,” which involves digital interactions between service users and peer support workers, who serve as lived experience experts to promote recovery.^4,5,19–21^ Digital peer support encompasses peer-delivered interventions through mobile applications, enabling communication between peer support workers and service users with and without real-time interaction via messages and video conversations.^5^ Digital peer support interventions for people with SMI can include, but are not limited to, resources to prevent relapse, strategies to manage mental health symptoms, enhancing physical health, improving medication adherence and physical activity engagement, and promoting self-management skills.^22^

Peer support has been identified as a key component of several digital interventions, enabling service users to connect with others facing similar challenges and as a means for mutual support.^21^ Digital peer support facilitates the exchange of health information and advice while enhancing user engagement with the digital tool.^2,23,24^ Integrating peer support into digital health interventions may improve effectiveness and clinical outcomes.^19^ Digital peer support interventions show promise for improving psychosocial outcomes, including hope, social support,^5^ service user autonomy,^20^ psychological well-being,^25^ and self-efficacy.^4,17^ They also strengthen social connections, providing opportunities for friendship and community engagement.^17,20,26,27^ Digital peer support interventions can significantly improve both mental and physical health,^16^ enhance selfhood and compassion while reducing symptoms of depression and anxiety.^25^

**Figure 1.**
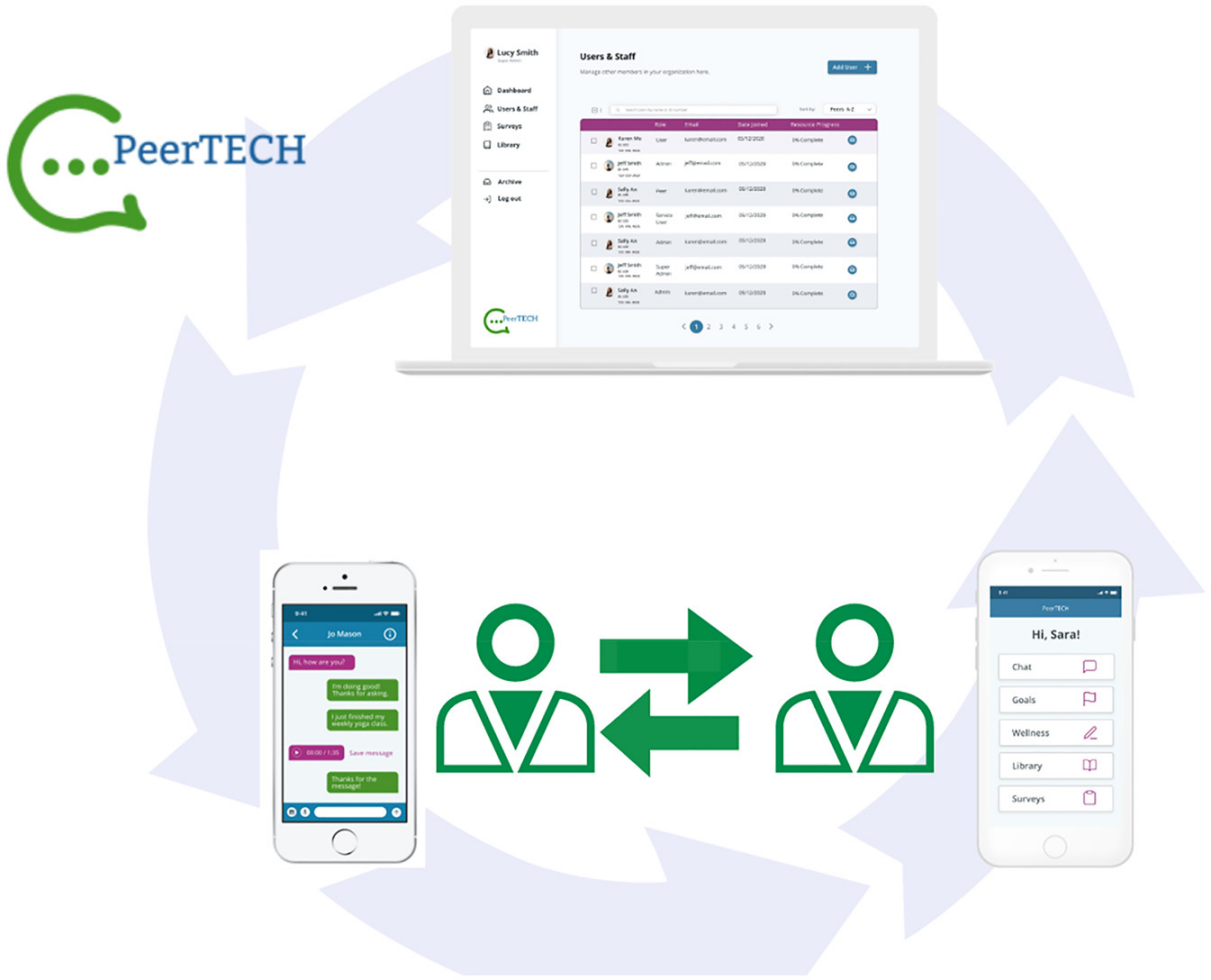
PeerTECH application.

The American PeerTECH application^28–30^ is a digital peer support intervention for people with SMI, designed to enhance self-management and goal-setting. As illustrated in Figure 1, the intervention combines mobile-accessible recovery content, weekly in-person sessions over ten weeks, and continuous in-app communication between peer support workers and users.

The system consists of two applications: one for users and one for peer support workers. The peer support worker application provides structured guidance for facilitating in-person sessions, assists with goal tracking and wellness planning, and enables messaging with assigned users. It also includes materials to support sharing lived experiences and promote self-management strategies.

The user application offers access to personalized goal setting, direct messaging with their assigned peer support worker, and a resource library containing ten structured mental, physical, and social health classes. These classes feature guided exercises and videos of people with SMI sharing personal experiences. Table 1 outlines the focus of each class.

**Table 1.** Focus of the PeerTECH classes.

Classes	The focus of the class
(1) Introductions, Smartphone Orientation, and Recovery and Health	Class 1 helps users access the application and explains peer support. It guides users in defining their recovery and how they want their life to become.
(2) Good Mental Health Starts With Good Physical Health and Social Health (vice versa)	This class explores the interplay between mental, physical, and social health. Users formulate personal goals to achieve during the intervention, with assistance from their peer support workers. Goals can be adjusted or maintained weekly. The class also covers creating action plans to help achieve these goals.
(3) Recovery is a Daily Process	Class 3 helps users create a wellness plan with resources to maintain happiness and health. Peer support workers assist in recognizing early warning signs, potential triggers, and coping strategies. Involvement of family and friends is encouraged, and collecting necessary contact information is essential. It also uses questions to promote self-reflection on the emotional and personal impact of pain or conditions, promoting understanding, acceptance, and a shift in perspective.
(4) How Stress Impacts Our Health	This class identifies factors impacting mental and physical health. It helps users recognize stressors and discomfort while highlighting ways to maintain happiness and reduce stress.
(5) Smoking and Living a Healthy Lifestyle	This class covers smoking-related health issues and the benefits of quitting, encouraging reflection on the pros and cons of smoking.
(6) Healthy Sleep	Class 6 emphasizes the importance of healthy sleep and the consequences of poor sleep and offers practical sleep advice.
(7) Dental Health	This class emphasizes the importance of regular dental visits, outlines when to see the dentist, and offers advice for maintaining good dental health.
(8) Exercise	Here, various strength exercises are demonstrated.
(9) Developing and Maintaining Relationships	This class focuses on social support, developing strategies to make new friends, and addressing loneliness to reduce stress and prevent social isolation. It also emphasizes the importance of asking for help to manage health problems and emotional challenges.
(10) Getting the Help You Want	This class focuses on navigating mental and physical healthcare when dealing with health issues. It covers how to access professionals, what to expect from healthcare in general, and how to make informed decisions about individual health.

**Figure 2.**
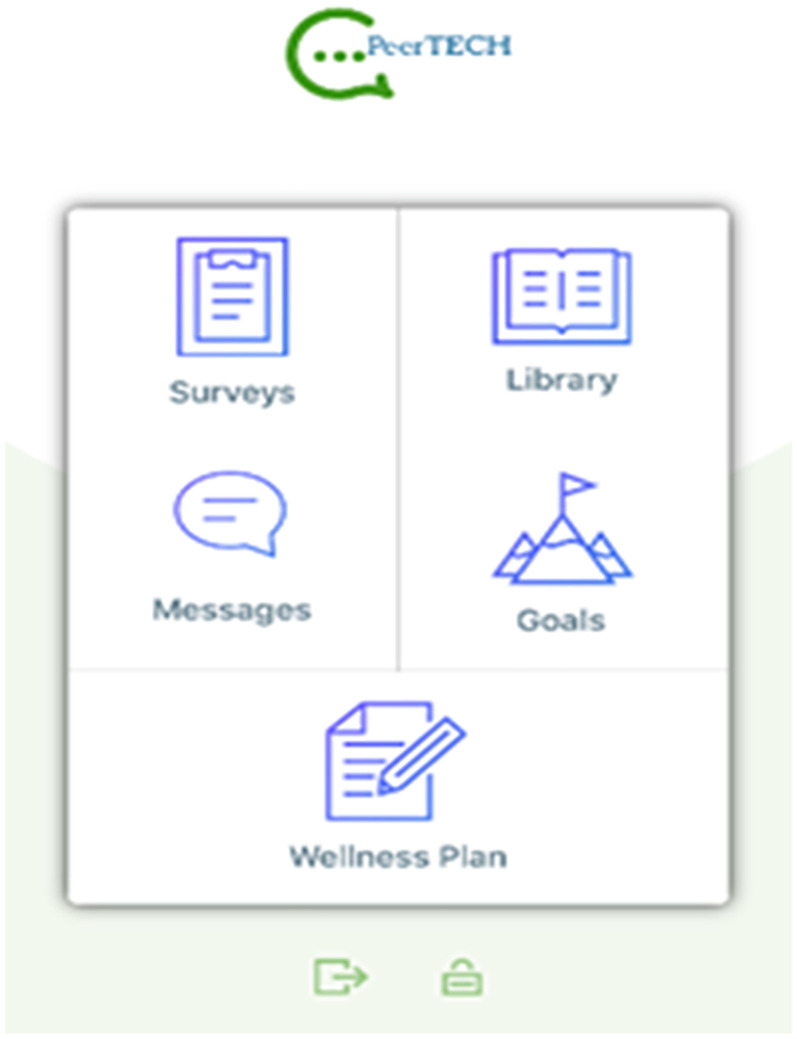
User home screen.

Upon logging into the app, users arrive at a home screen, where they can access the resource library, recent messages, their goals, and their wellness plan. Figure 2 displays the user's home screen.

Peer support workers send messages twice weekly to follow up on user progress, encourage, and offer goal-specific feedback. This routine interaction facilitates ongoing engagement between sessions and throughout the intervention.

Pilot studies in the United States indicate that PeerTECH enhances health literacy, promotes healthy behavior, and provides tools to prevent relapses, maintain health, and improve overall well-being.^28,30^ Storm et al.^31^ introduced PeerTECH to Norwegian service users, peer support workers, and professionals and conducted usability testing. Participants were found to be positive toward the application's content and considered it a relevant and user-friendly tool for supporting the self-management of their mental, physical, and social health.^31^ Thus, the next crucial step is adapting the application's content through co-creation to design a culturally responsive Norwegian version (which we named DigiPer, meaning digital peer) that ensures an optimal fit for the Norwegian context.^31^

Cultural adaptations of mental healthcare improve health and service outcomes for marginalized groups by engaging communities, understanding service users’ needs, and tailoring services accordingly.^32^ Key features include involving potential end users in needs assessment, adaptations, incorporation of cultural content, and implementation and delivery of services.^32^ Service user participation in the digital health development ensures that interventions align with the needs and expectations of end users, thereby enhancing acceptance, effectiveness, and uptake in the real world.^33^

Cultural adaptation of digital tools is challenged by complex socio-technical interactions between technology, users, and the wider system, where common issues include design and usability problems (e.g. login procedures), low motivation and engagement,^15,32,34^ digital inequality,^14,31^ and high attrition before intervention effects are achieved.^5^ Effective cultural adaptation of digital peer support interventions should address the unique needs and ethical considerations of people with SMI, including privacy, confidentiality, and information governance.^7,26,33,35^ These interventions must be meaningfully integrated into mental healthcare rather than treated as informal add-ons.^36^ Co-creation models can help address some of these unique challenges by ensuring that digital peer support tools are clear, usable, and engaging for people with SMI.^5,19,34^

Greenhalgh et al.^37^ ^(p. 39)^ state that co-creation is “the collaborative generation of knowledge by academics collaborating with stakeholders from other sectors.” Co-creation entails collaborative knowledge production, often centered on local or regional university-community partnerships, to align research and service development, thereby enhancing research and social impact.^37^ Co-creation can help address the persistent gap between research and its practical implementation.^37,38^ This approach involves collaboration throughout the implementation process, from initial stages such as commissioning and design.^39^ In mental health research, co-creation involves diverse stakeholders, including individuals with lived experiences of mental illness, professionals, and those who bring both perspectives.^39,40^ Participation in co-creation processes can promote personal growth and support career aspirations despite the challenges of mental illness, which is deemed particularly important for our study.^41^

Greenhalgh et al.^37^ explain that co-creation relies on three key success principles: (1) Adopting a systems perspective, which assumes emergence, local adaptation, and nonlinearity. By taking a systems perspective, the impact of the collaboration will be more robust and enduring, enhancing the user experience. (2) Framing research as a creative initiative centered on human experience, particularly that of service users and staff. (3) An appropriate leadership style is necessary to emphasize the importance of program framing, relationship dynamics, facilitation arrangements, interactions, sense-making, dialogue, and outcomes. Greenhalgh et al.^37^ emphasized that the co-creation of healthcare services should begin with the service user's experience, involve collaboration with staff to enhance the user experience, and ensure that the services are tailored to the experiences of service users and caregivers.

In Norway, the use of co-creation in mental health peer support has grown rapidly in recent years.^39,42^ However, applying co-creation to digital peer support interventions to culturally adapt them for individuals receiving mental healthcare is just beginning. In response to this research gap, this study aims to co-create and culturally adapt the DigiPer mobile application through workshops, videoconference demonstrations, and application testing with service users, peer support workers, and Norwegian community mental healthcare professionals. We use community mental healthcare when referring to services provided to people with SMI in the municipality. We sought to answer the following research questions:

1. How do community mental healthcare stakeholders perceive the content, classes, and functions of DigiPer?
2. What are their perceived challenges with DigiPer content and functions?
3. What are the perceived roles of peer support workers in DigiPer?

## Methods

### Study setting: Norwegian community mental healthcare

The Norwegian healthcare system comprises municipal health and care services and specialist health services.^43^ In Norway, healthcare services are publicly funded and accessible regardless of a person's socioeconomic status or geographical location.^43^ Municipalities offer mental and physical community-based healthcare, focusing on illness prevention, health promotion, treatment, care, and assistance with daily life.^44^ Municipalities are legally obligated to ensure access to general practitioners (GPs), who typically provide initial consultation and deliver ongoing healthcare.^44^ For people with SMI, municipal healthcare services can include, but are not limited to, supported housing with round-the-clock healthcare and mental health home care. For people with SMI who are receiving home-based healthcare services, the entitlement may also include free dental care provided through the public dental health service.^45^ Beyond the municipal scope, specialist health services are responsible for those with the most extensive mental healthcare needs, including inpatient and outpatient care through psychiatric hospitals and community mental health centers.^44^ In addition, multidisciplinary care models, such as Assertive Community Treatment (ACT) teams and Flexible Assertive Community Treatment (FACT) teams, have been established in various locations across Norway to provide continuous service accessibility for people with SMI.^46^

The current study was conducted with participants from community mental healthcare in two municipalities in western Norway, including one small and one large. The two municipalities differ regarding mental health service organization, resources, and technological maturity.

Workshops and application testing were conducted in the small Norwegian municipality with approximately 15,000 residents. In this municipality, community mental healthcare services are delivered by small, multidisciplinary teams. The services include home-based follow-up, low-threshold interventions, and individualized care planning. However, staff capacity and digital infrastructure appear relatively limited compared to larger municipalities, and integrated care models such as FACT are not implemented.

Additionally, videoconference demonstrations were held with participants from a larger Norwegian municipality with approximately 292,000 residents. This municipality offers a broader, more differentiated range of community mental healthcare services, including rapid access interventions, recovery-oriented programs, and structured housing options. While the municipality provides these services, the region collaborates with specialist health services to offer digital therapy for conditions such as anxiety and depression and integrated care models like FACT.

### Recruitment and participants

We used purposive sampling to recruit study participants eligible to contribute to the co-creation of the DigiPer mobile application.^47^ Service users, peer support workers, and professionals from community mental healthcare were considered stakeholders with the relevant knowledge and experiences necessary for this study. This diverse range of stakeholders aligns with the co-creation principles described by Greenhalgh et al.^37^

Inclusion criteria were defined for each stakeholder group to ensure relevance to the co-creation process. Service users were eligible if they had current or previous experience with mental illness. Peer support workers were qualified by having processed their own mental health experiences and using them to support others, and by being employed in a mental healthcare service. Professionals were included if they were employed in a community mental healthcare service.

Workshop and application testing participants were recruited through one service leader from community mental healthcare, whom MS contacted via e-mail with information about the study. The leader then provided eligible service users, peer support workers, and professionals with this information and selected participants based on their accessibility, interest, willingness, and experience with digital tools. In November 2022, we (JNS, HMHF, KLF, MS) attended the “Sterkere sammen” conference in Oslo, Norway, which focused on peer support. Interested participants were invited to a videoconference demonstration of DigiPer.

A final sample of 16 study participants was included. Workshops were scheduled with two service users, two peer support workers, and two professionals. Among them, five were female, and one was male. For the application testing, three service users, one peer support worker, and two professionals participated. Four of them were female, and two were male. Four peer support workers, two women and two men, were recruited to participate in these demonstrations.

### Research design

This study employs a qualitative participatory research design, well-suited for generating knowledge through the varied perspectives of participants at different stages of the research process.^47–49^ The ontological stance underlying this design views reality as multiple, subjective, and shaped by the stakeholders engaged in the research process, who both construct and influence it.^49^ Participatory research reduces the distance between researchers and study participants, facilitating interaction that allows knowledge to emerge through a co-creation process.^49^

### Co-creating a culturally adapted DigiPer

We use Greenhalgh et al.'s^37^ principles of co-creation to integrate the experiences and perceptions of key stakeholders in community mental healthcare, including service users, peer support workers, and professionals. To ensure that DigiPer was designed to meet the needs of service users and peer support workers in Norway, the mobile application was co-created and culturally adapted through a five-step process: (1) Translating PeerTECH, (2) Workshops, (3) Videoconference demonstrations, (4) Cultural adaptation of DigiPer, and (5) Testing DigiPer. Table 2 provides an overview of the co-creation process.

**Table 2.** The co-creation process to culturally adapt DigiPer.

Co-creation component	Step 1: Translating PeerTECH	Step 2: Workshops	Step 3: Videoconference demonstrations	Step 4: Cultural adaptation of DigiPer	**Step 5: Testing DigiPer**
Objective	To translate PeerTECH into Norwegian and culturally adapt it to the Norwegian context.	To engage stakeholders in reviewing DigiPer's translated content to ensure accuracy, and relevance to Norwegian mental healthcare, and users.	To present DigiPer to peers and gather feedback to enhance the application's relevance and usability for people with SMI.	To design a culturally responsive version of DigiPer for the Norwegian context, informed by feedback from stakeholders.	To evaluate DigiPer's usability, challenges, functionality, and content with potential end-users, and gather feedback for improvement based on lived experience perspectives.
Format	Conducted through professional translation combined with content and visual adaptation.	MS and HMHF led workshops with service users (N = 2), peer support workers (N = 2), and professionals (N = 2).	Individual video conference demonstrations with peer support workers (N = 4), led by MS.	Responsive development process informed by stakeholder input from workshops and videoconference demonstrations.	MS and JNS led testing sessions with service users (N = 3), peer support workers (N = 1), and professionals (N = 2).
Duration	Approximately 40 h across multiple working sessions.	3 h × 2 sessions.	30 min × 4 sessions.	Approximately 70 h across multiple working sessions.	3 h × 2 sessions.
Content	DigiPer uses the same interfaces and core functionalities as the PeerTECH application, serving as the basis for the translation. All English words at the user and peer interfaces (e.g. message systems, error messages, buttons, labels, images, symbols, and colors) were translated verbatim into Norwegian. This includes terms and words like *User* (Tjenestebruker), *Peer Specialist* *(*Likeperson), *Surveys* (Undersøkelser), *Library* (Bibliotek), *Messages* (Meldinger), *Goals* (Mål), and *Wellness Plan* (Velvære). In addition, images were replaced with culturally relevant Norwegian visuals. The resource library was initially translated verbatim and then adapted and updated with culturally responsive content adapted to the Norwegian context. Substantial updates were made to classes 7 and 10 to reflect the Norwegian healthcare system, including available services and users’ rights. In Class 7, we added information about free dental care rights, the importance of oral health, and tips for better dental hygiene. In Class 10, we added guidance on accessing healthcare, clarified the right to medical care, and explained the GP's role.	Application functionality demonstrations, content review, and open discussions. Participants reviewed DigiPer's interface and resource library. The first workshop covered classes 1–5 and 10, whereas the second reviewed 6–10. In both workshops, participants examined class 10 to optimize it for Norwegian users. Discussing task feasibility, health impact, and the role of American video content, highlighting preferences for user experiences.	Demonstration of DigiPer's interface, core features, and class structure. Open discussion to invite reflections and user-oriented feedback.	Revise content and perform ongoing testing. Replaced PeerTECH videos with animated videos aligned with DigiPer classes, covering: Class 1, What kind of life do you want, Class 2, Connection between mental, physical, and social health, Class 3, wellness planning, Class 5, benefits of quitting smoking, Class 7, oral health care, and Class 9, relationships. Added videos to: Class 4, relaxing body scan, Class 6, mindfulness sleep meditation, and Class 8, an exercise video.	Demonstration of DigiPer's system, installation, login, and core features. Testing of Class 1 and Class 6. Role-playing exercises of initial and continued interactions between users and peers. Review of Norwegian-language video content and participant evaluation of user experience.
Researchers’ role	Coordinated the translation process, ensuring linguistic, cultural, and systemic relevance.	Facilitated workshops, introduced the application, guided review, and collected participant feedback for cultural adaptation.	Led demonstrations and presented DigiPer, including its functions, content, and classes. Guided conversations to obtain feedback grounded in peer support practice.	Led adaptation efforts, including creating video scripts, testing DigiPer, checking cultural and linguistic accuracy, text and layout display, translation correctness, and technical functionality.	Facilitated sessions, presented DigiPer's functionality and key content features. Guided participants through installation and login processes, led role-play activities, group discussions, and feedback collection.
Study participants’ role	Participated in usability testing of PeerTECH, providing feedback that informed the translation and adaptation process. ^31^	Identified adaptation needs and shared feedback on language clarity, cultural relevance, meeting format, continued support, and user engagement strategies.	Offered feedback based on their peer support work experience, contributing to improvements in user experience and contextual relevance.	Provided feedback based on lived experience during workshops and demonstrations, contributing to content and technical refinements for improved cultural relevance.	Engaged in testing activities and role-play, provided feedback on usability, challenges, functionality, and content, reflected on their DigiPer experience and relevance to the Norwegian content.

### Data collection and analysis

Qualitative data were collected through workshops, videoconference demonstrations, and application testing to document stakeholders’ feedback and perceived challenges related to DigiPer's content, classes, and functions.^47,49^ The analyzed data material consists of written notes taken by MS and HMHF during the workshops, as well as notes written by MS during the videoconference demonstrations. The application testing was audio recorded and transcribed verbatim into 30 pages of written text by JNS, who translated the entire data material into English during the analysis process.

The qualitative data collected were analyzed using reflexive thematic analysis, which identifies meaning and patterns across the material, organizing results into rich, detailed themes.^50,51^ This consisted of six stages: (1) familiarizing with the data through repeated readings and initial coding ideas, (2) identifying and coding meaning units relevant to the study aim and research questions, (3) recognizing themes and subthemes and assigning meaning units accordingly, (4) critically evaluating themes to ensure they capture the entire data material, (5) refining and naming themes to shape the overall story the data tells, ensuring that our interpretations align with the data material and (6) writing the results section into five themes and ten subthemes, incorporating meaning units that reflect participants’ experiences and perceptions, study aim and research questions. In the results section, we present these themes and subthemes in detail.

Although the stages in the analytic process are described chronologically, the analysis continuously moved between the various stages of analysis before we agreed on the themes and subthemes presented. The collaborative efforts of authors from diverse educational and professional backgrounds strengthened the trustworthiness of the results. Our reflexivity and varied perspectives enriched the analysis and theme development, minimizing bias from a single researcher's preconceptions.^52^

### Ethics

The current study was approved by the Norwegian Agency for Shared Services in Education and Research (Sikt), which provides data protection services to ensure legal access to personal data. The project received approval under project number 769409, confirming its compliance with ethical guidelines and data protection regulations, including information security and privacy standards. This study followed the principles outlined in the Declaration of Helsinki.^53^ Participants received oral and written information about the study and provided written informed consent before data collection. Participation was voluntary, and participants were informed of their right to withdraw without giving a reason or facing consequences.

## Results

Below, we present the results from the co-creation process of the DigiPer mobile application. This includes feedback from community mental healthcare stakeholders, perceived challenges in application functionality, the roles of peer support workers, relevance in addressing day-to-day challenges, and the use of the application to promote a healthy lifestyle and a holistic view of health.

### Feedback and challenges in DigiPer functioning

#### Usability in DigiPer

The participants expressed their appreciation for the opportunity to test DigiPer, showing their willingness to adopt it and their belief in its usefulness for people with SMI. They highlighted the clear and organized presentation of essential topics, as well as its flexible usage and intuitive design, which allow users to navigate efficiently and access relevant, everyday content tailored to their needs.

Some individuals commented that “recovery” is frequently used in DigiPer. They suggested providing information about what “recovery” entails, as it might not be evident for Norwegian users. One said:

*Not everyone may be familiar with recovery. DigiPer mentions using your definition of recovery, but one should be familiar with the concept first.* (Service user from the workshop)

Videoconference demonstrations and testing revealed that the DigiPer classes could facilitate reflections, learning, and meaningful conversations between users and peers, particularly among those motivated to change. The participants emphasized the application's potential to be a resource and guide in daily life, offering innovative ideas and insights. They stressed the importance of clarifying its purpose during initial meetings between users and peers, as users often seek to understand its benefits, time commitment, and what the peer support workers can do to help.

With respect to DigiPer's suitability for other forms of treatment, opinions varied. While some recommended not implementing the DigiPer intervention under or immediately after treatment, others had a distinct perspective. One of them said:

*I was in therapy for a year, and it would have been helpful to have someone to talk to outside of the sessions. Over time, things can pile up, and forgetting what you want to say is easy. With a peer, you could address questions as they arise instead of waiting a week and potentially forgetting them.* (Service user from the application testing)

Concerns were raised if peer communication between weekly meetings was limited, which could hinder users from receiving timely assistance. During the testing, service users expressed worries about the postintervention period, as the 10-week duration might not be sufficient to achieve full recovery.

#### Managing complexity in DigiPer classes—Even small things can become overwhelming

During videoconference demonstrations and testing, concerns arose about whether the number of topics covered might discourage end users from completing the DigiPer intervention, as some classes included extensive information. Service users and professionals shared their experiences with previous digital follow-ups in municipalities, stating that they were too complex and had too many questions, leading to discontinuation. Peer support workers emphasized not overwhelming users, which could lead to a loss of motivation. One peer stated:

*I know that if there are too many things, things can quickly come to a standstill. Even small things can become overwhelming and create stress.* (Peer support worker at the application testing)

Class 1 was highlighted as containing particularly complex information, which was challenging despite its importance. Individuals who tested DigiPer also found Class 9 to have too much text, especially in the section about asking for help, discouraging them from reading. In contrast, classes 5, 6, and 7 were deemed essential and more focused, making them more accessible to engage with. The number of topics in each class must be determined to ensure usability.

All the study participants agreed that ten classes in DigiPer might be too many to address over 10 weeks. They emphasized the need for flexibility, allowing users to choose which classes and topics are important. For example, discussing smoking and dental health may be relevant, but others may find different topics more applicable. Overall, the content should remain manageable for the user group, avoiding excessive complexity that could hinder engagement.

### The peer support worker's role

#### Recognizing and addressing user challenges

Peer support workers found the peer role interesting. When testing DigiPer, they suggested that each peer support worker support three users throughout the intervention, considering that peer support workers typically have full-time jobs in addition to this role. One peer remarked:

*It would be nice to be a peer support worker in DigiPer. I have experience from various places, and I am familiar with the peer role. It is enriching to help others.* (Peer support worker from the application testing)

Several peer support workers highlighted the difficulty of fully understanding other people's challenges, even with personal experiences of mental illness. They explained that strategies effective for one person may not work for another, as individuals differ in how they invest their energy and manage their well-being. Peer support workers also stressed the importance of advancing their recovery and processing their experiences to support users’ challenges better. One service user illustrated this as follows:

*I have a job and try not to juggle too many responsibilities at once. I already have enough to manage independently, which can quickly become overwhelming. I have been sober for a few years now, but it is still easy to slip back.* (Service user from the application testing)

During the testing, the participants discussed the challenges that users might face at the beginning of the DigiPer intervention. They emphasized establishing a trusting relationship between the user and the peer. Peers at the videoconferences underlined the importance of meeting in person to discuss DigiPer content, classes, and goals, as direct interaction can promote openness and make users feel more comfortable sharing their experiences.

Service users stated that meeting others in a similar situation provides valuable support. They mentioned that peers of similar age and gender can better understand their needs and offer essential guidance during recovery.

However, several peer support workers provided feedback indicating a lack of clear role descriptions and undefined boundaries for the “peer” role. They suggested that DigiPer could be improved by offering a more detailed role description and clearer guidance for their work. Establishing clear boundaries around the peer support workers’ roles within DigiPer was essential to prevent them from becoming overwhelmed with responsibility and to ensure that the burden does not fall solely on them when users struggle. During the testing, a community where peers could meet to receive support, discuss issues, and share experiences was suggested. This would ensure that peer support workers have someone to contact in times of difficulty.

#### Balancing self-responsibility and support

Service users, peers, and professionals emphasized the importance of a balanced approach between self-responsibility and sufficient support during the DigiPer intervention. The workshop participants highlighted the need for users to address the positive and negative aspects of their lives. They also warned against feeling overwhelmed by too many changes and emphasized the need for patience in recovery. The professionals in the tests stressed the importance of asking users specific questions without putting too much pressure on them. A professional said:

*Many want immediate change, but we must also work on patience. If nothing has changed in two weeks, it is not that the intervention is not helping but that one may need more time. It can be easy to start things but maintaining them is difficult.* (Professional from the application testing)

In the videoconference demonstrations, peer support workers talked about how they perceived DigiPer to be effective in promoting change and recovery among certain users while acknowledging its lesser impact on others. They underscored that while it can be a valuable tool, users must actively contribute to their recovery. One peer support worker stated:

*DigiPer is not a miracle cure. No one can do the work for you. You must be willing to make changes in your life. This process may sometimes be uncomfortable, necessitating support to facilitate change.* (Peer support worker from the videoconference demonstrations)

Furthermore, during videoconference demonstrations, peer support workers emphasized that people may have encountered past challenges that hinder their ability to embrace new opportunities. While DigiPer may seem promising in theory, translating it into practice presents challenges that often result in frustration. In such instances, peers emphasize the importance of helping users identify individual stressors, breaking them down, focusing on positive aspects, and making tasks more manageable.

### Content of DigiPer: Addressing day-to-day challenges

#### Supporting personal growth and goal setting

All groups of study participants highlighted the challenges individuals with SMI face in managing their daily lives. Providing efficient mental and physical healthcare for these individuals can be difficult. DigiPer could offer valuable structure and advice to help people with SMI achieve their desired life.

The workshop participants appreciated the self-reflection questions in class 3, which allowed users to explore difficult past experiences and work toward understanding and accepting their condition. They recognized that their life experiences had provided valuable lessons and shaped them, expressing hope for the future and acknowledging that everyone can achieve their goals with proper support.

On the other hand, when testing class 2, the participants answered questions such as “What life do you want?” and “What have you always dreamed of doing?” to be broad and challenging to answer. It could also be challenging to address these questions during class 2, as service users and peer support workers may not yet be well-acquainted. Despite this, the participants acknowledged the relevance of these questions and appreciated the opportunity to articulate their aspirations and set personal goals. One service user expressed the complexity of the task by stating:

*It is not easy to answer what kind of life I desire. At the very least, it should be a better life than I had. I want a life where I excel socially and treat myself kindly. That is what I desire. A peaceful life.* (Service user from the application testing)

Service users had encountered similar questions in treatment contexts, particularly when planning for the future, including potential reentry into the workforce. One of them felt that he had progressed, stating:

*I have a job and a stable and meaningful life. Therefore, such questions are no longer crucial. It is not my primary objective; it addresses the minor aspects of achieving a fulfilled life. Currently, loneliness and social challenges are the primary struggles.* (Service user from the application testing)

Regarding Class 2, workshop participants recommended avoiding unrealistic goals, as these could exacerbate users’ situations. They suggested that formulating achievable personal goals and subgoals to combat negative thoughts and enhance well-being can help users complete the intervention. The participants appreciated the emphasis of the application on celebrating small victories, daily progress toward manageable goals, and the ability to set new goals afterward.

#### Digiper as a tool to build supportive networks

All the participant groups emphasized the crucial role of social relationships and networks in maintaining stable health. They discussed how DigiPer could be a tool for helping users build supportive networks. The participants highlighted the importance of providing information about community resources and meeting places for network building, such as exercise facilities and activity centers. They expressed concern about avoiding people who negatively influence them and instead connecting with people who understand their experiences and emotions, allowing them to be genuine and receive meaningful support. One peer noted:

*When you are struggling, you feel like you have no one - using the network maps in Class 1 makes it visible that you have people around you, dispelling the feeling of isolation.* (Peer support worker from the videoconference demonstrations)

Furthermore, workshop participants highlighted the exercises in Class 9 as potential boosters of social skills and network development. These exercises emphasize connecting with others, engaging in interactions, and initiating activities. The “You and I” exercise resonated positively among the participants. However, it was acknowledged that these exercises may not suit everyone's needs and preferences.

### Utilize DigiPer to encourage a healthy lifestyle

#### Addressing substance use and smoking habits

The study participants had differing opinions on whether substance use should be a focus in DigiPer. While workshop participants saw value in addressing this issue, service users who partook in the testing felt that it was acceptable to avoid questions about substance use. One of them expressed:

*It is good to have a place where you do not have to talk about substance use. It can lead to feelings of guilt and disengagement. With DigiPer, there are enough other topics to focus on. You do not need to use substances if all the points are covered.* (Service user from the application testing)

Peer support workers from the videoconference demonstrations suggested that alcohol or substance usebe incorporated into Class 5, “Smoking and Living a Healthy Lifestyle,” as addiction is a common issue. Furthermore, peer support workers and service users acknowledged that not everyone necessarily wants to quit smoking and recommended avoiding attempts to stop smoking during high-stress periods and other challenges, as smoking can have a calming effect.

Moreover, participants raised concerns about Class 5's primary focus on quitting smoking during the testing of DigiPer. They emphasized that a healthy lifestyle encompasses more than smoking cessation, limiting DigiPer's potential to lead to broader changes in health behavior. One service user said:

*I do not see anything about a healthy lifestyle in this class, just a lot about smoking. Then, you are left wondering what constitutes a healthy lifestyle. It is more than just smoking or not smoking. Diet is the most essential component of everyday life. It can be something people struggle with, such as eating junk food.* (Service user from the application testing)

#### Improving sleep and stress management

Several study participants noted that many people with SMI struggle with a lack of sleep or oversleep. Professionals who tested DigiPer explained that despite attending sleep courses, applying advice remains difficult, leading service users to question the purpose of consistent sleep routines when sleep remains elusive. Breaking the cycle of daytime napping after sleepless nights was considered burdensome, especially without daytime work or meaningful activities. A focus on sleep can sometimes irritate, as one service user shared:

*I would feel stressed if someone told me what to do. Just relax, and I would be all wound up. When you start having trouble sleeping for consecutive nights, stress sets in. A lack of sleep leads to anxiety and negative thoughts about oneself and others, creating a real mess.* (Service user from the application testing)

Despite this, the participants believed that the sleep advice provided in class 6 could be relevant. They suggested that users go through the list step by step, and they might discover something they have not tried before. Perhaps combining multiple strategies over time and being patient is necessary.

The workshop participants found value in both the reading and listening tasks in class 4, which could help them stay calm. Specifically, they highlighted that the listening tasks were particularly well-suited for individuals with dyslexia. At the workshop and during testing of DigiPer, participants acknowledged that stress could have both negative and positive effects on the body. Some stress levels were always present, and they agreed to live with it. One peer stated:

*Breathing is crucial for stress management. I struggle to breathe using my stomach; it only reaches my chest. As a result, my neck becomes stiff, and my body feels locked.* (Peer support worker from the application testing)

### A holistic view of health

#### Connecting users to relevant services

The workshop participants emphasized the importance of DigiPer in connecting users to healthcare, dental care, and social services, including NAV (Norwegian Labor and Welfare Administration). While satisfied with Class 2, which comprehensively addresses mental, physical, and social health, all study participant groups highlighted challenges in collaborating with health and social services. Service users noted a lack of information exchange between community and specialist health services, leading to uncertainty about whom to contact when struggling and how to be referred to specialist health services. During a videoconference demonstration, a peer specified the following:

*Collaborating with health and social services can be demanding. It may entail interactions with hospitals, community mental health centers, GPs, legal guardians, and NAV.* (Peer support worker from the videoconference demonstrations)

Service users often contact GPs for healthcare but face barriers such as reluctance or communication difficulties that can lead to untreated pain and deteriorating health conditions. Workshop participants appreciated the continuity of community mental healthcare but faced challenges with their inability to adjust medications. They valued care coordination meetings and suggested better outreach during crises. They also noted that people with SMI may not always recognize the efforts of professionals such as GPs, psychologists, social educators, and nurses when they feel bad. Service users in the workshops suggested that having someone reach out during challenging times or acute needs would be beneficial. The study participants were encouraged to use the wellness plan in Class 3 to input information about assigned services and whom to contact when needed.

In Class 7, workshop participants found information about dental health crucial, particularly regarding their legal entitlement to free dental treatment in Norway. However, they also experienced challenges in determining eligibility for this treatment, citing dental anxiety and financial constraints as reasons some individuals avoid seeking dental care. Additionally, the participants suggested including information about the right to choose treatment from specialist health services in DigiPer.

#### A need for support to overcome financial challenges

All the study participants reported that finances could create challenges for people with SMI, making it difficult to meet their social needs. People can struggle to prioritize their dental health and exercise if they cannot pay their bills, stay awake worrying about financial matters, or are unsure if they have a safe place to live. The study participants emphasized that DigiPer lacked a focus on finances. A service user remarked:

*Finance is a major stressor that affects many aspects of life, especially those in or seeking treatment. I see people rushing into treatment today. Without proper financial support, people may continue to struggle even after treatment. If they had received financial help during treatment, their problems might not have been so large.* (Service user from the application testing)

The participants also noted that individuals with SMI often rely on income from NAV, but accessing NAV proves difficult through in-person visits, digital means, or phone calls. This lack of communication leaves service users without funds during weekends, exacerbating their frustration. They can easily express anger, leading to disconnections and blocks from NAV. Consequently, they turn to the study participants for assistance, highlighting their challenges as a third party in relaying messages. One service user said:

*It is not always easy for peer support workers to advise on everything, but if you are a peer, you have gone through those experiences and can recommend whom to contact. For instance, NAV can be contacted for financial matters.* (Service user from the application testing)

## Discussion

In this qualitative participatory study, we co-created and culturally adapted the DigiPer mobile application to the Norwegian context. We tested its function and classes with service users, peer support workers, and professionals from community mental healthcare. The participants perceived DigiPer as having proper usability and found its focus, classes, and content helpful. Moreover, the participants perceived the role of the peer support worker in DigiPer to be meaningful. However, maintaining a clear boundary in such a role when delivering peer support remains challenging. Building a trusting relationship with service users and among peer support workers, as well as encouraging a healthy lifestyle, connecting to relevant services, and addressing financial barriers, was highlighted as significant by all participants. A more user-centered joint effort and holistic recovery approach were expected to be realized in DigiPer.

In DigiPer, co-creation entails bringing together voices from those with lived experience, especially peer support workers with successful recovery experiences and professionals.^37^ By providing a dedicated communication platform, our results showed that DigiPer promotes open dialogue and mutual learning between peers and service users, supporting Fortuna et al.'s theoretical model of “reciprocal accountability”^4^ and the findings of Åkerblom and Ness^39^ on how peer support workers help promote genuine and balanced partnerships.

We found that the co-creation approach^37^ makes DigiPer particularly valuable for vulnerable and traditionally marginalized groups, who often lack a voice in the Norwegian mental healthcare system. Our results revealed many subtle and underlying stressors experienced by people with SMI, such as housing and financial constraints, dental anxiety, and mistrust and confusion toward health and social care systems. These challenges often remain “hidden” behind individual struggles, leading to unfair labels such as “mentally unstable” rather than recognizing people as holistic individuals with complex life histories.^54^ The co-creation approach in this study empowers service users to actively contribute to their recovery plans in DigiPer, which are more sensitive to individual needs and circumstances.^4^ Our findings indicate that DigiPeer's ability to connect users with relevant services was desirable among participants. Research^14,15^ further supports this by highlighting how digital peer support can help reduce disparities in healthcare access for people with SMI by facilitating their connection with essential services. Additionally, our results emphasize that DigiPer should allow service users to have a peer of the same age and gender, as this enhances mutual understanding of their needs, which is consistent with Andalibi and Flood's^27^ findings on that connecting individual based on shared identities rather than diagnoses is beneficial, as it can help reduce stigma and raise their voices.

Although DigiPer appears to be a promising tool, our peer support workers voiced concerns about establishing clear boundaries and responsibilities with the delivery of peer support services, which is consistent with results from early studies.^55–57^ Peer support workers can add value and complement traditional mental healthcare through their lived experience with SMI.^4^ However, unlike professionals in mental healthcare, peer support workers often navigate a “dual relationship” with their service users, which may include interactions beyond designated support programs, such as after-hours communication.^57,58^ While blurred work-life boundaries can help service users achieve specific goals,^59,60^ they also risk having a negative emotional impact on peer support workers, potentially contributing to burnout, work-life conflict, and a drain on one's motivation.^59,61,62^ As this can be a foreseeable challenge for a peer-led, digital technology-enhanced tool like DigiPer, our study participants suggested a detailed role description and clear guidance for their work. Based on this finding, we advocate that clearly defined role descriptions are vital for successfully implementing digital peer support,^19^ as better boundary management can foster co-creation in real-world practice and between peer support workers and service users.^39^ One boundary management strategy involves integrating DigiPer into peer support work routines by establishing clear communication goals between peer support workers and service users early on. For example, peer support workers can use chat functions to expand access to hard-to-reach groups through geographical barriers, while integrating regular physical meetings with service users to allow for service flexibility. Another approach we suggest is to enforce clear worktime boundaries, such as limiting chat availability to working hours, except for urgent life events such as suicide risk, where emergency interventions, such as contacting GPs, will be taken.^63,64^

When using technology to deliver peer support, it is essential to consider how this new delivery approach impacts the mental and physical well-being of peer support workers themselves, such as burnout or trauma triggers. This is a major concern expressed by our participants in the study, as it aligns with previous studies that emphasize the importance of safeguarding the health of peer support workers when implementing peer support interventions.^58,65^ Hence, building strong connections within the peer community is essential to strengthening mutual support throughout the implementation of digital health technology programs such as DigiPer.^66^ Proactively offering training and supervision is one way to connect peers to mitigate potential risks and scale up the effectiveness of the intervention.^67^ Effective peer support work relies on training to equip peer support workers with the necessary skills to maintain professional and ethical boundaries, alongside structured supervision offering peer workers a space for reflection, problem-solving, and support.^16,19,36^ For example, providing simulation-based training before deploying a digital health technology program can clarify the program's goal and help peer support workers better coordinate with service users.^1^ An early study by Fortuna et al.^68^ revealed that simulation training increased peer support workers’ capacity to provide support through the technology program. Additionally, regular supervision and interactions among peer support workers while using digital health technology programs such as DigiPer can facilitate the exchange of user experience and challenges, promoting program fidelity and proper use of the technology.^68,69^

Compared with community mental health services in the United States, Norwegian community mental healthcare involves multiple stakeholders and complex factors that make the implementation of digital health tools challenging, especially for the most vulnerable groups.^70^ To ensure successful cultural adaptation, digital solutions tailored to people with SMI, addressing their distinct needs and challenges, will be essential to maintaining accessibility and feasibility.^32^ Future studies should consider synthesized and implementation-oriented theoretical frameworks, such as Greenhalgh and Abimbola's NASSS (Non-adoption, Abandonment, Scale-up, Spread, and Sustainability) framework^71^ when assessing the accessibility and feasibility of DigiPer. The role of peer support workers is still relatively new in Norway. While there is potential for peer support communities to benefit from using DigiPer, proper training and supervision on accessibility and digital literacy must be established. Additionally, in this instance, simulation could be used to develop and promote adequate training, supervision, and digital literacy for peer support workers.^1^ Future studies could also explore the social impact of adopting digital health technology programs such as DigiPer for peer support communities and service users and compare Norway and the United States. We suggest that future findings could be valuable for better mental health peer support guidelines in Norway.

### Strengths and limitations

A notable strength of the current study was the inclusion of several key stakeholders from community mental healthcare. This participatory approach facilitated meaningful interactions that helped capture diverse perspectives, ensured that Digiper aligned with the Norwegian mental healthcare context, and strengthened the transferability of the findings to other settings.^47,52^ Using the co-creation principles^37^ ensured that the development of Digiper was influenced by relevant stakeholders, strengthening the relevance of the results to practice.^47,49^ Moreover, collecting data from multiple sources, including workshops, videoconference demonstrations, and application testing, helped obtain comprehensive perceptions regarding DigiPer, strengthening the credibility of the findings.^47^

Our qualitative participatory research design^47–49^ and purposive sampling ensured that relevant participants were included. However, this approach may have introduced selection bias, as participants were chosen based on accessibility, interest, and willingness to participate.^72^ Their interest could have influenced participants’ feedback on the intervention and experience with digital tools. Another limitation of the study is the small sample size, which may limit the transferability of findings to broader populations when interpreting the results.^47^ Additionally, although we continuously tested the mobile application, the testing period may not capture long-term usability issues. Thus, extended use over time could identify additional recommendations for refinements. To conclude, although efforts have been made to ensure accurate translations, nuances that could affect the application's effectiveness may still be lost in translation.

## Conclusion

In this qualitative participatory study, we co-created and culturally adapted the DigiPer mobile application and tested its functionality with Norwegian service users, peer support workers, and community mental healthcare professionals. This study contributes to the growing field of digital peer support in Norwegian mental healthcare, emphasizing the importance of co-creation, usability, and structured implementation in interventions like DigiPer. Beyond addressing the research gap regarding how digital peer support interventions for people with SMI can be co-created and culturally adapted to other contexts, this study provides valuable insights into DigiPer's usability, where all participant groups perceive its culturally adapted content and classes as relevant. Input from these key stakeholders helped ensure the relevance of DigiPer to the Norwegian context and the health needs of people with SMI.

Additionally, this study provided relevant general knowledge about important aspects to consider when implementing digital tools in community healthcare and promoting self-management for people with SMI and others with health needs. By highlighting the use of co-creation principles, usability, and implementation, this research contributes to ongoing efforts to refine digital peer support interventions in Norway.

## Supplemental Material

sj-docx-1-dhj-10.1177_20552076251380648 - Supplemental material for Co-creating DigiPer mobile application: A digital peer support tool for managing mental, physical, and social health in serious mental illness Supplemental material, sj-docx-1-dhj-10.1177_20552076251380648 for Co-creating DigiPer mobile application: A digital peer support tool for managing mental, physical, and social health in serious mental illness by Jorunn Nærland Skjærpe, Hilde Marie Hunsbedt Fjellså, Karen L Fortuna, Bo Wang and Marianne Storm in DIGITAL HEALTH
